# Supplementary material for: Classification and Identification of Industrial Gases Based on Electronic Nose Technology
Source: Sensors (Basel). 2019 Nov 18;19(22):5033. doi: 10.3390/s19225033 (PMC6891334; doi:10.3390/s19225033)
Supplement: Supplementary file 1 [file sensors-19-05033-s001.pdf]

Supplementary Materials (including four figures)

Classification and Identification of Industrial Gases Based on Electronic Nose Technology

Supplementary Materials 1: The results of four industrial gases classification and identification by PCA

During each sampling period, the response data were collected from 10 sensors of the PEN3 E-nose. We obtained 100 training sample groups. The steady-state response value occurs between the 45th and 57th seconds of the sensor response. The mean value is the average response data value of the sensor's 120 seconds sampling time. The number of sample points in the steady-state response value was 13 times that of the mean value.

Figure S1 shows classification results of the PCA algorithm under the steady-state value.

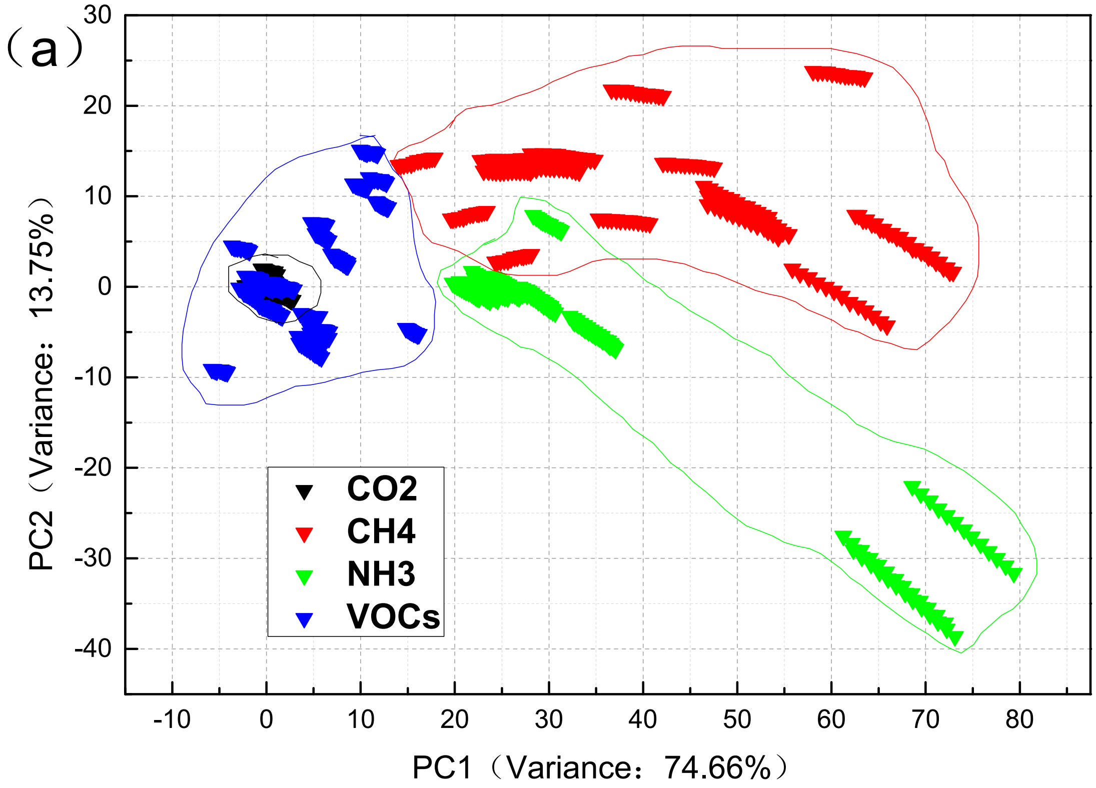

Figure S1. The PCA classification results of the four industrial gas samples under the steady-state response value.

Supplementary Materials 2: The results of the classification and identification of four industrial gases using LDA

Figure S2 shows classification result of the LDA algorithm under the steady-state value.

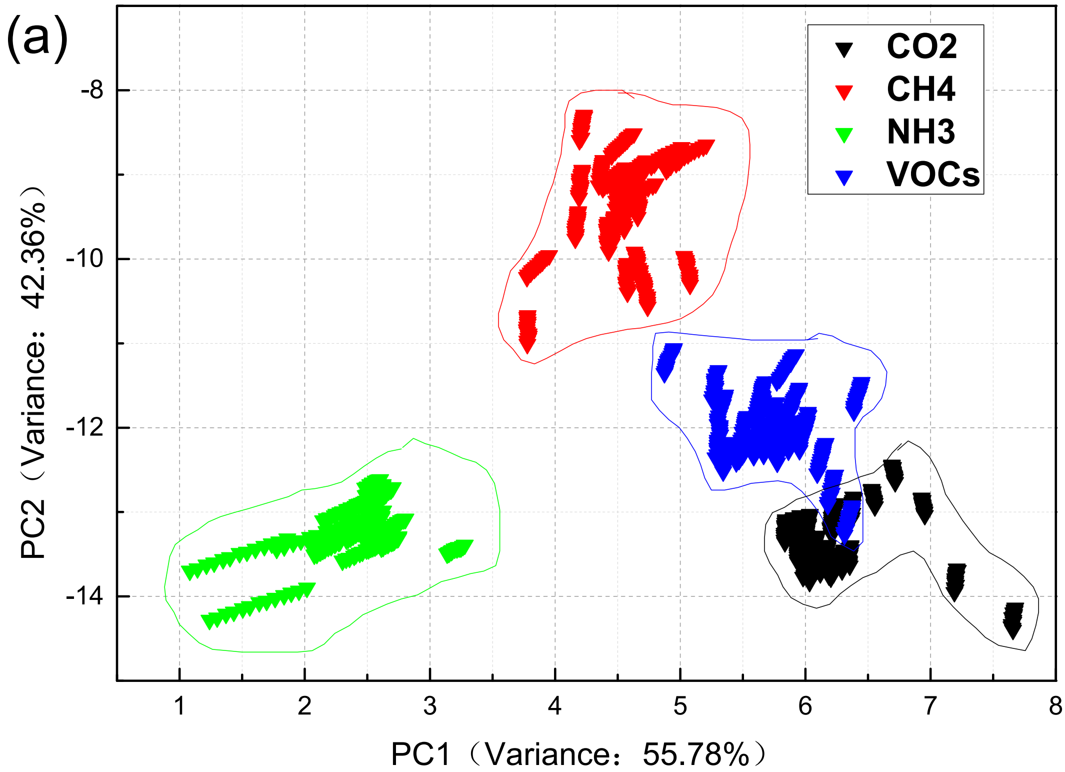

Figure S2. The LDA classification results of the four industrial gas samples under steady-state response value.

Supplementary Materials 3: The results of the classification and identification of four industrial gases using PCA + LDA

Figure S3 shows classification result of the PCA + LDA algorithms under the steady-state value.

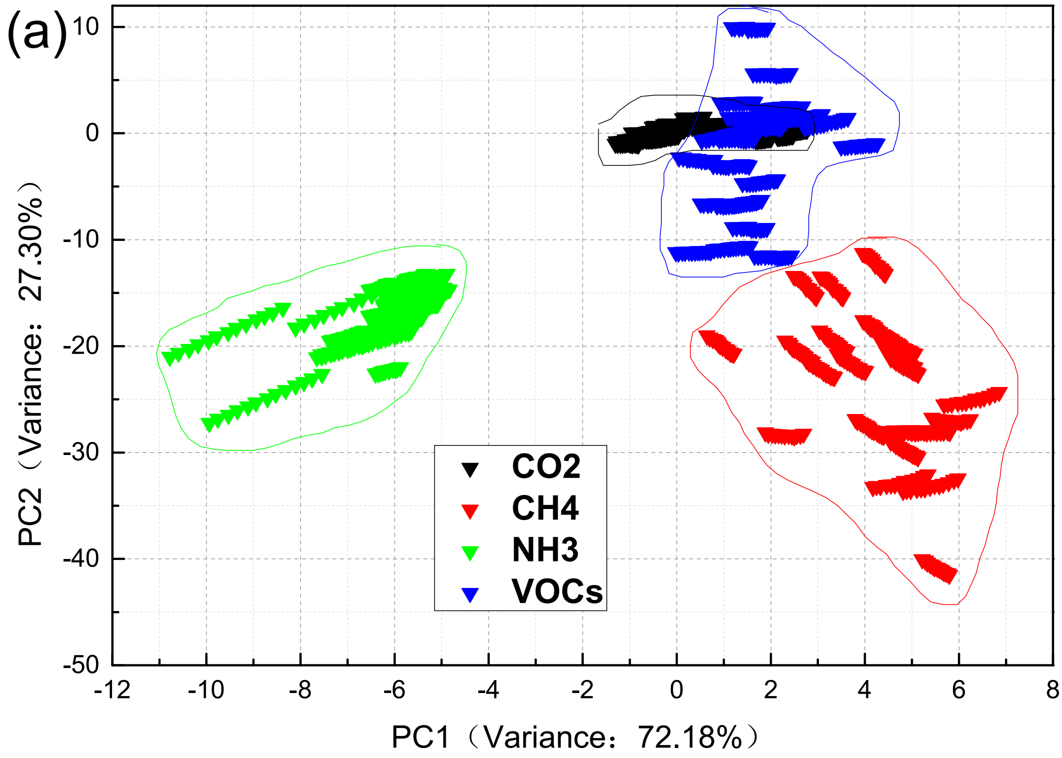

Figure S3. The PCA + LDA classification results of the four industrial gas samples under steady-state response value.

Supplementary Materials 4: The experimental setup for specific concentration gas samples

In our paper, we obtained the specific concentration gas samples using a gas distribution device (GDD). The gases were fluxed alone from the gas channels. We used air as carrier gas during the experiment. Air was used to clean the GDD and PEN3 during the experiment. The effect of the carrier gas (air) on E-nose response was negligible and so it was ignored. We obtained different concentrations of gas through the GDD.

As shown in Figure S4, the GDD has five input channels and one output channel. We obtained different concentrations of gas samples via the LCD touch screen. From the screen of GDD, we controlled and debugged the input of the gas sample, fine-tuning the mass flow controller for higher precision. By slowly opening the valve of the gas cylinder, the gas samples were fed into the GDD through a hose. The gas sample was thoroughly mixed and diluted, and the specific concentration of gas was obtained. The E-nose collected the gas information from the output of the GDD through a hose. The E-nose data port was connected to the computer USB, and the gas information was saved to the computer for research and analysis purposes.

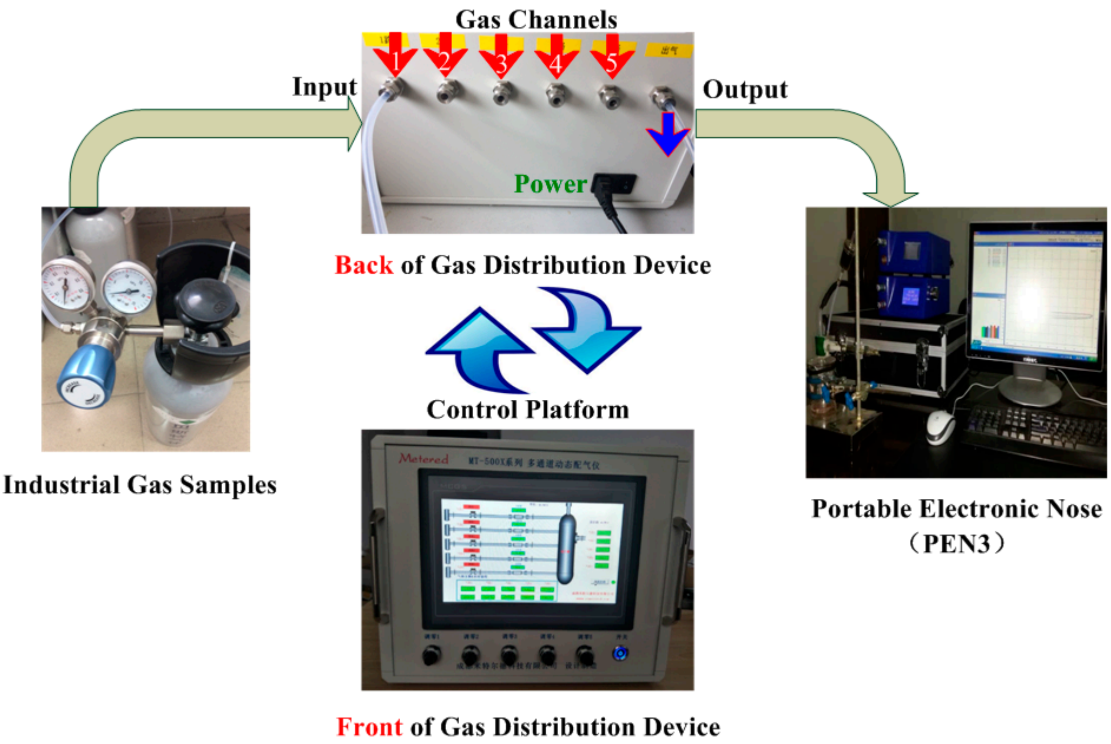

Figure S4. Experimental equipment and processes for obtaining specific concentrations of gas samples.
